# Supplementary material for: Utilization of archived neonatal dried blood spots for genome-wide genotyping
Source: PLoS One. 2020 Feb 21;15(2):e0229352. doi: 10.1371/journal.pone.0229352 (PMC7034898; doi:10.1371/journal.pone.0229352)
Supplement: S1 Table — (DOCX) [file pone.0229352.s001.docx]

**S1 Table. Associations between residual DBS characteristics and storage environment among DBS archived at the Michigan Neonatal Biobank between 1992 and 2008.**

| **Residual DBS characteristics** | **Uncontrolled environment^1^**  **(n = 101)** | **Controlled environment^2^**  **(n = 298)** | **p-val^3^** |
| --- | --- | --- | --- |
| **Leukemia status, n (%)** |  |  | 0.93 |
| Leukemia | 6 (6%) | 17 (6%) |  |
| No leukemia | 95 (94%) | 281 (94%) |  |
| **Infant sex, n (%)** |  |  | 0.97 |
| Male | 52 (51%) | 154 (52%) |  |
| Female | 49 (49%) | 144 (48%) |  |
| **Maternal race/ethnicity, n (%)** |  |  | 0.89 |
| Non-Hispanic White | 79 (78%) | 229 (77%) |  |
| Non-Hispanic Black | 14 (14%) | 43 (14%) |  |
| Non-Hispanic Asian | 4 (4%) | 9 (3%) |  |
| Hispanic | 4 (4%) | 17 (6%) |  |
| **Total DNA yield in µg, median (range)** | 5.5 (2.3-13.3) | 5.2 (2.4-14.4) | 0.05 |
| **260/280 measurement, median (range)** | 1.5 (1.4-1.7) | 1.6 (1.5-1.7) | <0.001 |
| **Call rate %, median (range)** | 99.3 (80.4-99.9) | 99.9 (93.8-99.9) | <0.001 |

^1^Uncontrolled temperature, 35% humidity

^2^70 degrees Fahrenheit, 35% humidity

^3^Pearson chi-square, Fisher’s exact, or Wilcoxon rank-sum p-value comparing residual DBS characteristics by storage environment
