# Supplementary material for: Utilization of archived neonatal dried blood spots for genome-wide genotyping
Source: PLoS One. 2020 Feb 21;15(2):e0229352. doi: 10.1371/journal.pone.0229352 (PMC7034898; doi:10.1371/journal.pone.0229352)
Supplement: S2 Table — (DOCX) [file pone.0229352.s002.docx]

**S2 Table. Counts of residual DBS sample call rate, storage environment, and collection period by genotyping array position among DBS archived at the Michigan Neonatal Biobank between 1992 and 2008.**

|  | **Genotyping array row position** | | | | | | | | | | | | **p-val^1^** |
| --- | --- | --- | --- | --- | --- | --- | --- | --- | --- | --- | --- | --- | --- |
|  | **1** | **2** | **3** | **4** | **5** | **6** | **7** | **8** | **9** | **10** | **11** | **12** |  |
| **Sample call rate** |  |  |  |  |  |  |  |  |  |  |  |  | 0.92 |
| ≥ 99.0% | 22 | 27 | 29 | 30 | 28 | 29 | 27 | 31 | 29 | 31 | 28 | 28 |  |
| < 99.0% | 7 | 6 | 5 | 4 | 6 | 5 | 6 | 3 | 4 | 3 | 5 | 6 |  |
| **Storage environment** |  |  |  |  |  |  |  |  |  |  |  |  | 0.99 |
| Uncontrolled environment | 21 | 25 | 25 | 26 | 25 | 26 | 24 | 26 | 24 | 26 | 24 | 26 |  |
| Controlled environment | 8 | 8 | 9 | 8 | 9 | 8 | 9 | 8 | 9 | 8 | 9 | 8 |  |
| **Uncontrolled environment** |  |  |  |  |  |  |  |  |  |  |  |  | 0.86 |
| Sample call rate  ≥ 99.0% | 4 | 4 | 5 | 5 | 3 | 4 | 3 | 6 | 5 | 6 | 5 | 4 |  |
| Sample call rate  < 99.0% | 4 | 4 | 4 | 3 | 6 | 4 | 6 | 2 | 4 | 2 | 4 | 4 |  |
| **Controlled environment** |  |  |  |  |  |  |  |  |  |  |  |  | 0.62 |
| Sample call rate  ≥ 99.0% | 18 | 23 | 24 | 25 | 25 | 25 | 24 | 25 | 24 | 25 | 23 | 24 |  |
| Sample call rate  < 99.0% | 3 | 2 | 1 | 1 | 0 | 1 | 0 | 1 | 0 | 1 | 1 | 2 |  |
| **DBS collection period** |  |  |  |  |  |  |  |  |  |  |  |  | 0.99 |
| 1992-1995 | 8 | 8 | 9 | 8 | 9 | 8 | 9 | 8 | 9 | 8 | 9 | 8 |  |
| 1996-1999 | 8 | 9 | 9 | 9 | 9 | 9 | 9 | 9 | 8 | 10 | 8 | 9 |  |
| 2000-2003 | 5 | 8 | 7 | 7 | 7 | 7 | 7 | 7 | 8 | 7 | 8 | 8 |  |
| 2004-2008 | 8 | 8 | 9 | 10 | 9 | 10 | 8 | 10 | 8 | 9 | 8 | 9 |  |

^1^Pearson chi-square or Fisher’s exact p-value comparing residual DBS call rate, storage environment, collection period by genotyping array position.
