# Supplementary figures and images for: Utilization of archived neonatal dried blood spots for genome-wide genotyping
Source: PLoS One. 2020 Feb 21;15(2):e0229352. doi: 10.1371/journal.pone.0229352 (PMC7034898; doi:10.1371/journal.pone.0229352)

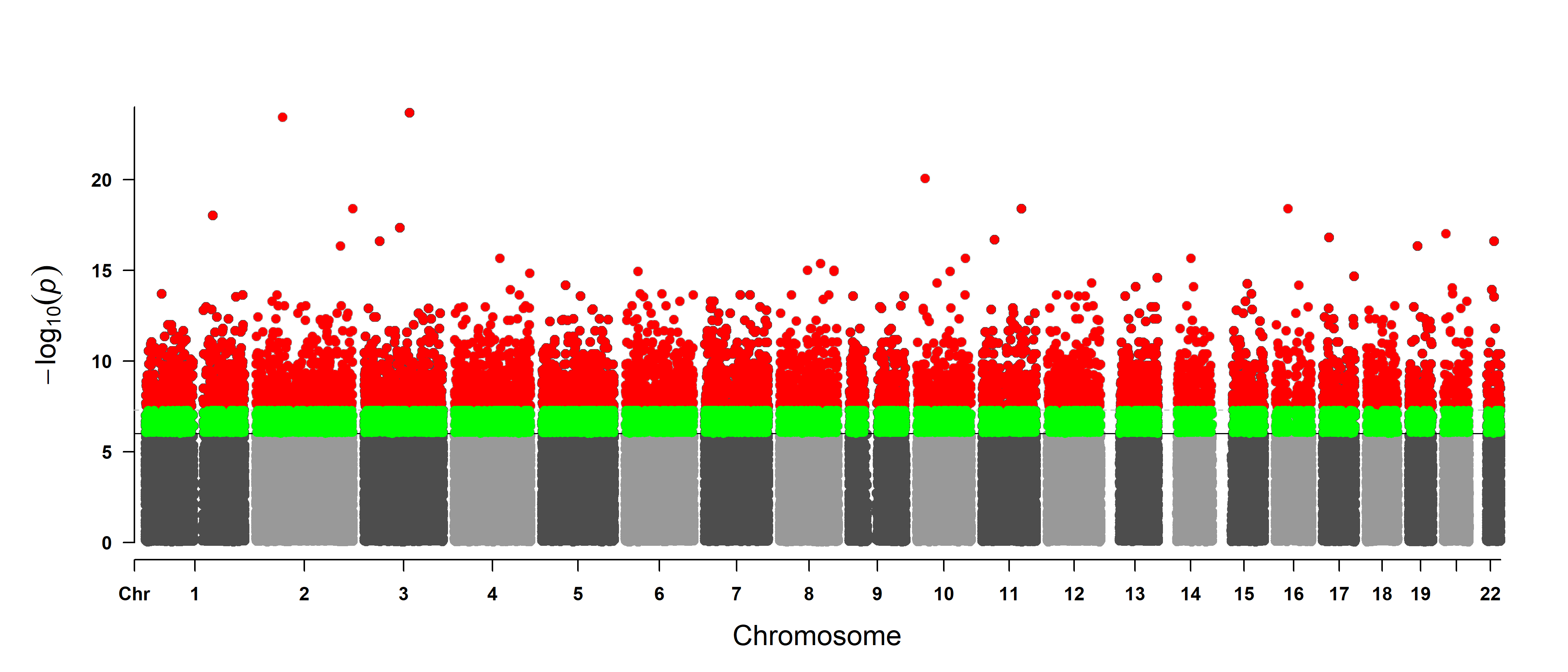

Supplement: S1 Fig — Black and red horizontal lines signify p-value ≤ 1×10^−5^ and ≤ 5×10^−8^, respectively. (TIFF) [file pone.0229352.s003.tiff]
